# Variation of Aflatoxin Levels in Stored Edible Seed and Oil Samples and Risk Assessment in the Local Population

**DOI:** 10.3390/toxins14090642

**Published:** 2022-09-17

**Authors:** Shahzad Zafar Iqbal, Muhammad Waqas, Ahmad Faizal Abdull Razis, Sunusi Usman, Nada Basheir Ali, Muhammad Rafique Asi

**Affiliations:** 1 Department of Applied Chemistry, Government College University Faisalabad, Faisalabad 38000, Pakistan; 2 Department of Food Science, Faculty of Food Science and Technology, Universiti Putra Malaysia, Serdang 43400, Malaysia; 3 Natural Medicines and Products Research Laboratory, Institute of Bioscience, Universiti Putra Malaysia, Serdang 43400, Malaysia; 4 Laboratory of Food Security and Food Integrity (FOSFI), Institute of Tropical Agriculture and Food Security, Universiti Putra Malaysia, Serdang 43400, Malaysia; 5 Food Toxicology Lab, Nuclear Institute for Agriculture & Biology, Faisalabad 38950, Pakistan

**Keywords:** selected seeds and oils, aflatoxins, storage effect, dietary assessment

## Abstract

Five hundred and twenty samples of edible seeds and oilseeds (sunflower, palm, peanut, sesame, cotton, and grapeseed) were purchased from markets, farmers, and superstores in the central cities of Punjab, Pakistan. A total of 125 (48.1%) edible seed samples from a 6 ≤ months storage period, and 127 (48.8%) from a 2 ≥ years storage period were found to be infested with AFs. The average elevated amount of AFB_1_ and total AFs was observed in a 2 ≥ years storage period, i.e., 28.6 ± 4.5 and 51.3 ± 10.4 µg/kg, respectively, in sesame seeds. The minimum amount of AFB_1_ and total AFs was observed in palm seed samples with a storage period of 6 ≤ months, i.e., 9.96 ± 2.4, and 11.7 ± 1.90 µg/kg, respectively. The maximum amount of AFB_1_ and total AFs were observed in peanut oil samples, i.e., 21.43 ± 2.60 and 25.96 ± 4.30 µg/kg, respectively, with a storage period of 2 ≥ years. Therefore, the maximum dietary intake of 59.60 ng/kg/day was observed in oil samples stored at a ≥ 2 years storage period. The results of the present study concluded that a significant difference was found in the amounts of total AFs in edible seed samples stored at 6 ≤ months and 2 ≥ years storage periods (*p* < 0.05).

## 1. Introduction

In a recent survey, it was determined that 690 million people (8.9% of the global population) are facing hunger and lack of food, and this number increases by 10 million people/year [1]. Food insecurity affects 1.3 billion people, with 21.3% of children younger than five years being termed as underdeveloped. The situation has become more serious with the current COVID-19 pandemic, leaving an estimated 83 to 321 million people undernourished. In another estimation, the world will need to produce 60% more food to feed the population of around 9.3 billion in 2050 [1]. Pakistan is an agricultural country; however, it still cannot produce sufficient edible oils for domestic requirements. The consumption of 27.73 million metric tons of vegetable oils per capita, with a total of 19.5 kg for edible and inedible purposes, has been recorded. It has been estimated that 18 to 20% of calories are achieved from edible oils, with a total average intake of 2400 calories per day. Over the last decade, the consumption of liquid cooking oil increased over the consumption of solid fats [2]. By importing palm oil from Indonesia and Malaysia, Pakistan meets 80–90% of its total edible oil demand. The imported palm oil is used in various goods, such as chocolates, vanaspati ghee, soap, and bakery goods [3]. However, the seeds used to produce other oils may be affected by environmental conditions developed during the pre-harvest or post-harvest cultivation of crops. Furthermore, drying, transportation, and storage conditions also play a vital role in fungal attacks [4,5].

Mycotoxins are secondary metabolites which produce a wide range of toxins from fungi under specific climatic and storage conditions [6,7,8]. The most toxic type of mycotoxins are aflatoxins (AFs) [9]. These are produced by filamentous fungi such as *Aspergillus flavus*, *Aspergillus parasiticus*, and *Aspergillus nomious* [10]. Aflatoxin B_1_ (AFB_1_) has been classified as group 1 (carcinogenic to humans) by the International Agency for Research on Cancer [11]. It has shown carcinogenic and cytotoxic effects [12]. The impact of AFs toxicity in animals and humans has been observed in previous studies. The exposure of AFs in animals or humans could come from direct inhalation or contact, or by consuming contaminated food from plants or animals [13]. The toxic effects of AFB1 are digestive tract disorders, growth retardation, liver toxicity, or even cancer [14,15]. Besides these carcinogenic effects, other reports have shown its mutagenic and immunosuppressive effects in animals [6]. In animals, pulmonary toxicity has been observed in vivo in male albino rats [16], genotoxicity has been observed in vivo in mice [17], and gastrointestinal toxicity has been observed in vivo in rats [18], pigs [19], and chickens [20,21]. In addition, several studies from around the world [22,23,24,25,26] and from Pakistan [5,27] have documented the presence of AFs in edible seeds and oil samples.

Considering the above circumstances, our study is focused on investigating the presence of AFs in selected edible seed and oil samples stored for various storage periods. Furthermore, the dietary intake of AFs in individuals from different age groups consuming edible oil has also been evaluated. Therefore, this study will help food agencies to implement strict regulations for AFs in edible seed and oil samples.

## 2. Results and Discussion 

### 2.1. HPLC Method Validation

Linearity, repeatability, reproducibility, recovery analysis, detection limits (LOD), and the limit of quantification (LOQ) were important parameters of the analytical method. The precision of the method was analyzed by adding 3 fortified amounts of AFB_1_, AFG_1_, AFB_2_, and AFG_2_ (1, 4, and 8 µg/L), in a mixed sample of edible oils. The average recovery values varied from 74.5 to 96.5%, with the relative standard deviation (RSD) from 9 to 21.5%. For linearity, seven-point standard curves were constructed for each Afs, i.e., for AFB_1_ and AFG_1_ (0.50, 6, 14, 50, 140, 250 µg/kg) and AFB_2_ and AFG_2_ (1, 5, 20, 40, 60, and 80 µg/kg). The linearity of the curves could be assessed with the value of the coefficient of determination (R^2^) ≥ 0.99. The detection limits (LOD) of AFB_1_ and AFG_1_ were 0.08 µg/kg, and LOQ was 0.24 µg/kg. However, the LOD and LOQ for AFG_2_ and AFB_2_ were 0.09 and 0.27 µg/kg, respectively. In the previous study, the linearity range for AFB_1_ and AFG_1_ was 1 to 80 µg/kg and 0.5 to 12 µg/L for AFB_2_ and AFG_2_. The LOD and LOQ were 0.04 and 0.12 µg/kg for AFB_1_ and AFG_1_ and 0.6 and 0.18 µg/kg for AFG_2_ and AFB_2_, respectively [5]. The results agreed with those in the study by Waqas et al. [27].

### 2.2. Occurrence of Afs in Inedible Seeds and Oil Samples

The study focused on examining the amount of AFB1 and total AFs in stored edible seeds (6 ≤ months and 2 ≥ years) in 520 samples from the central and southern cities of Punjab, Pakistan. The findings indicated that 125 (48.1%) samples of selected edible seeds from a 6 ≤ months storage period, and 127 (48.8%) samples from a 2 ≥ years of storage period were detected to be contaminated with AFs (levels ≥ LOD). The extreme mean value of AFB_1_ and total AFs were 28.6 ± 4.5 and 51.3 ± 10.4 µg/kg in the sesame samples (2 ≥ years storage period), respectively, as presented in Table 1.

**Figure 1:**
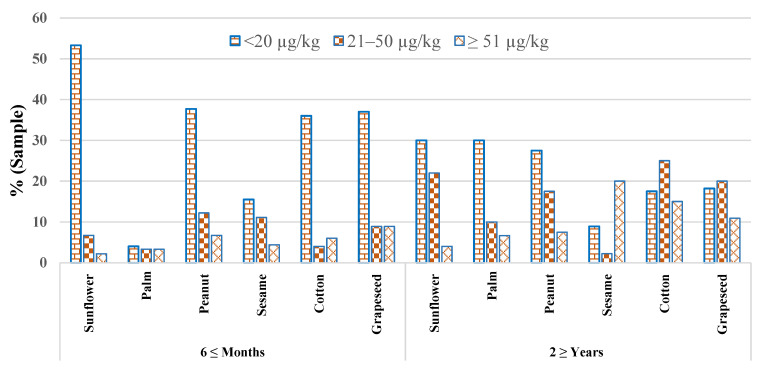
Graph showing the percentage of total aflatoxins in stored selected edible seed samples.

However, the minimum amount of 9.96 ± 2.4 and 11.7 ± 1.90 µg/kg was documented for AFB_1_ and total AFs in palm seeds samples (2 ≥ years storage period), respectively, as shown in Table 2. Furthermore, the amounts of total AFs (<20 µg/kg, 21–50 µg/kg, and ≥51 µg/kg) in selected edible seed samples from different storage periods are represented in Figure 1. The results show that a higher percentage of total AFs levels was observed in edible seed samples stored for a 2 ≥ year storage period.

**Figure 2:**
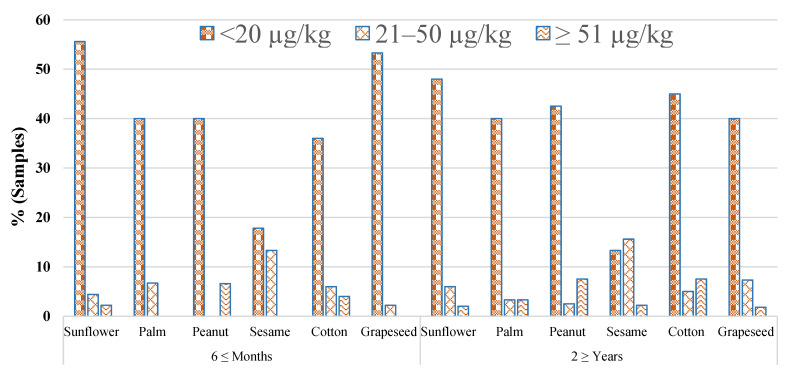
Graph showing the percentage of total aflatoxins in selected stored edible oil samples.

Samples of 520 edible oils, 260 samples each from a 6 ≤ months storage period and a 2 ≥ years storage period, were examined for the prevalence of AFB_1_ and total AFs, as indicated in Table 2. Similarly, the levels of total AFs in different ranges from both storage periods are represented in Figure 2. Samples of 125 (48.1%) from a 6 ≤ months storage period and 132 (50.1%) samples originating from a 2 ≥ years storage period were found to be confirmed with AFs. The highest means of AFB_1_ (21.43 ± 2.60 µg/kg) and total AFs (25.96 ± 4.30 µg/kg were demonstrated for the 2 ≥ years storage period for palm samples, and the minimum amount of 6.25 ± 3.20, and 6.48 ± 4.30 µg/kg were recorded for grapeseed samples from a 6 ≤ months storage period, respectively. There existed a significant difference in the amounts of AFs between the 6 ≤ months and 2 ≥ years storage periods (*p* < 0.005).

In a previous study, Yeboah et al. [28] documented aflatoxin levels in groundnut seeds during storage in Ghana. Aflatoxins were detected after four months of storage only in Nkosour (148.21 µg/kg), while *Adepa and Kwame Danso* verified higher amounts of AFB_1_ (45.918 µg/kg), and B_2_ (410.974 µg/kg), respectively. Razis et al. [5] studied 779 samples of edible nuts from southern Punjab (Pakistan) and recorded 20.9 ± 3.10 µg/kg total AFs in seedless watermelon seed samples. However, 15.9 ± 3.60 µg/kg of total AFs was recorded in seedless melon seed samples. Wenndt et al. [7] documented 595 samples of cereals, pulses, and oilseeds, analyzing AFB_1_ contaminations and their health risks. Ogungbemile et al. [8] studied aflatoxins in cowpea seeds in Nigeria. They recorded elevated amounts of aflatoxins B_1_ (1.5 × 10^−2^ μg/g), G_1_ (0.60 × 10^−2^ μg/g), G_2_ (1.0 × 10^−2^ μg/g), and B_2_ (0.80 × 10^−2^ μg/g), respectively.

However, in previous studies, Mohammed et al. [29] analyzed unrefined (*n* = 21) and refined (*n* = 40) samples of sunflowers seeds. They observed that 6 (15%) out of 40 samples were contaminated with AFB1, ranging from LOD to 218 ng/g. Furthermore, 3 samples contained amounts higher than the limit according to the Tanzanian Bureau of Standards (TBS) and the European Commission/European Union (EC/EU) permissible limit (2 ng/g). In another study, Banu and Muthumary [24], from Karnataka, India, studied sunflower oil samples and observed that 10 (43.4%) samples out of 23 were infested with AFB_1_, while all refined oil samples had levels ˂ LOD. Beheshti and Asadi [30], from Iran, investigated the incidence of AFs in sunflower and safflower seeds and revealed that 64% of the sunflower seed samples were infected with AFs. The findings revealed that 103 (83.7%) samples of safflower seeds (mean amounts 2.81 to 0.44 ng/g), and 8 (16%) samples of sunflower seeds (mean level 40.68 ng/g) were found to be contaminated. The levels of AFB_1_ in 5 sunflower and 2 and safflower seed samples were elevated above the recommended regulations of the European Union (2 ng/g). Ferrracane et al. [25], from Italy and Morocco, documented that 3 (10%) of 30 olive oil samples were polluted with AFB_1_ and OTA, varying between 0.54 to 2.50 ng/g. In another study, Karunarathna et al. [22] analyzed 59 vegetable oil samples (43 branded and 16 unbranded) and concluded that a considerable amount (37.5%) of samples were positive for AFs and AFB_1_ within the range of 2.25 to 72.70 μg/kg, and 1.76 to 60.92 μg/kg, respectively. AFB_1_ levels in 2 oil samples were observed in amounts higher than those recommended by the EU (2 μg/kg). Mariod and Idris [26], from Sudan, studied 241 groundnut samples (186) and sunflower (55) oils, and the results showed that the growing, harvesting, and storage of crops were the main reasons for the high contamination of AFs.

The findings revealed that 14.5% of sunflower oil and 54.8% of groundnuts samples had levels of AFs ≥ LOD. Nabizadeh et al. [31] examined the AFs (AFB_1_, B_2_, G_1_, G_2_) in six categories (canola, blend, frying, olive, sunflower, unrefined olive oil) of 97 edible oils, and results revealed that 98% of the samples had an AFB_1_ level ˂ LOD. Some contaminated samples had AFs levels that were within the standard established by EU regulation (2 μg/kg for AFB_1_ and 4 µg/kg for total AFs). Shar et al. [32], from Pakistan, investigated cotton seeds and cottonseed cakes for the incidence of AFB_1_ in 110 samples and observed that the extreme mean amount of AFs in cottonseed cakes was 89 μg/kg.

Earlier studies have shown that mycelia and aflatoxigenic fungi could already be present in harvested grains. The primary carrier for the fungal attack might be insects [33,34]. During postharvest, the dominant effects are the length and method of storage [35,36]. Therefore, the quality of stored food depends on the storage conditions and methods [37]. Furthermore, the methods and storage times might differ depending on the geographical region [38]. The dominant factors regarding geographical impact in Pakistan are illiteracy and using old traditional methods of cultivation, harvesting, and storage [39]. The cost and availability of storage units are also vital in maintaining the quality and safety of food products [36]. Therefore, continued monitoring of mycotoxins in foodstuffs helps to establish models that predict the seasonal variation of AFs in different food products [40].

Furthermore, drought during preharvest or postharvest might affect the crops and provide favorable conditions for the growth of fungi such as *Aspergillus* [41,42,43]. The difference in the levels of AFs in seeds might be influenced by factors such as harvesting practices, storage conditions, transportation, the use of analysis techniques, etc. [44].

## 3. Dietary Assessment of Aflatoxins in Edible Oil Samples

The dietary intake of total AFs in edible oil (sunflower oil) was analyzed in male individuals from central and southern Punjab, as described in Table 3. Sunflower oil is utilized during cooking in Pakistan; therefore, we have estimated dietary intake using sunflower oil samples. The highest mean dietary intake of 59.60 ng/kg/day was estimated in oil samples stored for a 2 ≥ years storage period in individuals ≥33 years old.

In previous studies, a dietary intake of 0.90 µg/kg/day was detected for a sunflower oil sample in female individuals aged between 16–22 years old [42]. A dietary intake of 6.30 µg/kg/day was estimated for pumpkin seed samples in female individuals from Pakistan [5]. Assessing dietary intake levels depends on many factors, such as eating habits, traditions, genetic variations in the human body, seasonal variations, regional differences, and ethical beliefs [27].

## 4. Conclusions

The study examined the variation in the levels of AFs in edible seeds and oil samples stored for different storage periods (i.e., 6 ≤ months and 2 ≥ years storage periods). The research has shown that significant differences were detected in the levels of AFs in edible seeds from different storage periods (*p* < 0.05). However, a non-significant difference was observed in the amounts of AFs in edible oil samples from different storage periods (*p* ≥ 0.05). A considerably high level of dietary intake was observed in sunflower oil. The results confirm the continuing importance of monitoring AFs in edible seeds, and strict regulations should be imposed to avoid/minimize their presence in edible seeds.

## 5. Methodology

### 5.1. Sampling

The samples (*n* = 520) of edible seeds (sunflower, palm, peanut, sesame, cotton, and grapeseed) were purchased from markets and superstores (at different storage conditions, i.e., 6 ≤ months and 2 ≥ years) from the central cities of Punjab, Pakistan. Next, the oil was obtained from each sample, and their I.D.s were marked accordingly. A total of 260 samples of each edible seeds were collected from 6 ≤ months and 2 ≥ years of storage. In Pakistan, the cultivation season of edible seeds is January–February, and the harvesting season is May–June. Therefore, a 6-month storage period means that the crop was harvested in June 2021. However, for a 2-year storage period, the cultivation period of the crop was the June 2019 season. The edible seeds were mostly stored in jute bags, and the storage temperature was room temperature. The sample size of each seed was not less than 5 kg each. The methodology for collecting seed samples was random, and the gross sampling technique was used for lab samples.

### 5.2. Chemicals and Reagents

The chemicals, including AFs standards, HPLC grade methanol, acetonitrile, n-hexane, chloroform, sodium chloride, and anhydrous sodium sulfate, were purchased from Sigma-Aldrich, (Steinheim, Germany). Dichloromethane, trifluoroacetic acid (TFA), and other chemicals were obtained from Sigma-Aldrich (Karachi, Pakistan). Furthermore, of Milli-Q^®^ EQ 7000 (Merck, Darmstadt, Germany) distilled water (double distilled) was used during the research.

### 5.3. Edible Seed Samples and Aflatoxins Extraction

The extraction process for AFs in edible seed samples was achieved, as discussed in the methods of [45]. Briefly, the sample (25 g) was mixed in 125 mL of 55% methanol solution. Then 100 mL hexane, with the addition of 2 g of NaCl, was added and homogenized for 15 min with an orbital shaker. Next, the solution was filtered (Whatman No.1, Merck, Darmstadt, Germany), and the solution was placed in a dark place for 30 min to develop polar and non-polar phases. From the polar phase, a portion of 25 mL solution was moved into the separating funnel, and 10 mL of chloroform was added. The process was repeated 3 times to completely extract the AFs from the mixture. Finally, the chloroform layer was drained out from the two layers in a 250 mL beaker using anhydrous sodium sulfate. A water bath evaporated the solution at 60 °C until dry.

### 5.4. Oil Samples and Extraction of Aflatoxins

The process for extracting the AFs from the edible oil samples was conducted as described by AOAC [45], with some modifications. First, a sample (50 mL) of oil was mixed in a solution of 250 mL of 55% methanol. Next, the solution was centrifuged (4500 rpm) for 5 min after adding 50 mL of 0.1N HCL solution. The solution was filtered (Whatman no 1), and 50 mL of the solution, along with 50 mL each of hexane and 10% solution of NaCl were moved in a separating funnel. The mixture was mixed vigorously for 60 s and allowed to develop polar and non-polar phases. Then, the polar layer was drained out in a separator, and 20 mL of dichloromethane was added, and the mixture was shaken vigorously. After 5 min, the dichloromethane layer was moved into a vial and evaporated to dryness. Next, the derivatization of AFB_1_ and AFG_1_ was carried out by adding 100 µL of TFA to the dried oil or seed samples and vortexed (30 s). The residues were left in a dark place for 5 min. Finally, 400 µL of acetonitrile–water (1:9 *v*/*v*) solution was added, and a 20 µL sample was subjected to HPLC study.

### 5.5. HPLC Conditions

The Shimadzu (Model-LC-10A, Kyoto, Japan) HPLC instrument with a stationary phase (C18 column; 250 mm × 4.6 mm, 5 µm of Discovery, HS, Bellefonte, PA, USA) and a detector (fluorescence; RF-530, Kyoto, Japan) were used in a reverse phase mode. The HPLC mobile phase was composed of acetonitrile, water, and acetic acid (10; 50; 40 *v*/*v*/*v*) with a 1 mL/min flow rate during analysis. The emission and excitation wavelengths were set at 325 and 295 nm, respectively.

### 5.6. Dietary Intake Assessment

The assessment of daily intake was done using the method described in [46,47], where daily intake (EDI) is calculated as:

$$\mathrm{Dietary}\;\mathrm{intake}\;(µg/\mathrm{kg}/\mathrm{day})=\frac{Daily\;Consumption\;of\;oil\;\left(kg/day\right)\times Mean\;total\;AFs\;\left(µg/kg\right)}{Individual\;mean\;weight\;\left(kg\right)}$$

**Figure 3:**
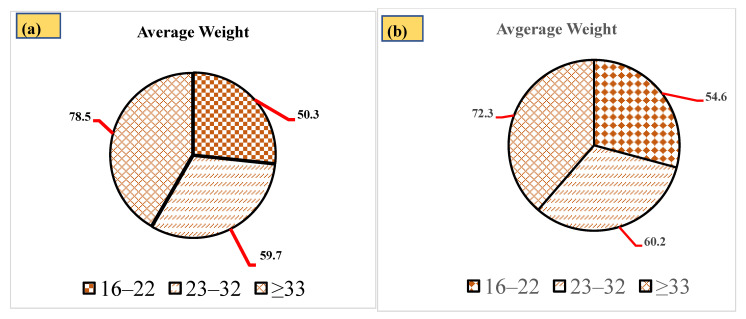
Average weight of different age groups from a (**a**) central Punjab, and (**b**) Southern Punjab population.

The daily intake data was attained by designing a food frequency questionnaire for 600 participants, of which 405 replied. The different age groups of individuals from central Punjab and Southern Punjab, along with their average weight, are shown in Figure 3a,b, respectively. Prior written consent was obtained, with the condition that their information would not be disclosed. All ethical and confidential guidelines have been implemented during the evaluation of the food frequency questionnaire.

### 5.7. Statistical Analysis

The experimental observations and results were expressed as the mean ± standard deviation. The seven-point calibration curve was constructed for each AFs, and the value of R^2^ was evaluating correlation/regression analysis. Linearity was determined with a coefficient of determination and straight-line equations. The significant difference in the amounts of AFs in stored seeds and oil samples was evaluated using a one-way Analysis of Variance (α = 0.05) SPSS (IBM 26, Chicago, IL, USA). The significant differences among treatments were calculated using LSD analysis.

## Figures and Tables

**Table 1:** Occurrence of aflatoxin B_1_ and total aflatoxins (µg/kg) in selected edible seeds available in markets.

Sample Category	6 ≤ Months Storage Period					2 ≥ Years Storage Period				
	Samples	Positive	Mean AFB_1_	Mean AFs	Range Total AFs	Sample	Positive	Mean AFB_1_	Mean AFs	Range Total AFs
	*n*	*n* (%)	µg/kg	µg/kg	µg/kg	*n*	*n* (%)	µg/kg	µg/kg	µg/kg
Sunflower	45	28 (62.2)	11.9 ± 2.5	14.3 ± 1.80 **	LOD-98.6	50	28 (56.0)	15.1 ± 3.4	22.5 ± 6.5 **	LOD-112.5
Palm	30	14 (46.6)	9.96 ± 2.4	11.7 ± 1.90 **	LOD-65.5	30	14 (46.6)	16.5 ± 6.5	25.8 ± 7.4 **	LOD-110.5
Peanut	45	21 (46.6)	18.24 ± 3.4	20.9 ± 3.70 **	LOD-98.6	40	21 (52.5)	28.2 ± 8.5	36.4 ± 9.1 **	LOD-170.8
Sesame	45	14 (31.1)	22.1 ± 2.5	24.6 ± 4.50 **	LOD-60.5	45	14 (31.1)	28.6 ± 4.5	51.3 ± 10.4 **	LOD-75.5
Cotton	50	23 (46.0)	23.6 ± 4.5	25.3 ± 5.60 **	LOD-125.8	40	23 (57.5)	26.2 ± 6.5	41.9 ± 9.4 **	LOD-145.5
Grapeseed	45	25 (55.5)	24.0 ± 4.6	29.1 ± 7.50 **	LOD-175.5	55	27 (49.0)	27.8 ± 7.6	45.4 ± 11.3 **	LOD-155.75
Total	260	125 (48.1)				260	127 (48.8)			

**: the differences in aflatoxins levels between storage periods in edible seeds were significant (*p* < 0.05).

**Table 2:** Occurrence of aflatoxin B_1_ and total aflatoxins in selected edible oil samples.

Sample Category	6 ≤ Months Storage Period					2 ≥ Years Storage Period				
	Sample	Positive Samples	Mean AFB_1_	Mean AFs	Range Total AFs	Samples	Positive Samples	Mean AFB_1_	Mean AFs	Range Total AFs
	*n*	*n* (%)	µg/kg	µg/kg	µg/kg	*n*	*n* (%)	µg/kg	µg/kg	µg/kg
Sunflower	45	28 (62.2)	9.19 ± 2.10 ^N.S^	10.81 ± 2.40 ^N.S^	LOD-78.5	50	28 (56.0)	11.96 ± 2.40	13.70 ± 2.50 ^N.S^	LOD-95.5
Palm	30	14 (46.6)	7.80 ± 1.70 **	7.90 ± 3.15 **	LOD-44.5	30	14 (46.6)	12.20 ± 3.20 **	13.4 ± 3.90 **	LOD-75.5
Peanut	45	21 (46.6)	13.32 ± 2.70 **	15.00 ± 3.20 **	LOD-70.5	40	21 (52.5)	21.43 ± 2.60 **	25.96 ± 4.30 **	LOD-150.5
Sesame	45	14 (31.1)	18.77 ± 3.20 ^NS^	20.10 ± 3.50 ^N.S^	LOD-42.9	45	14 (31.1)	21.66 ± 4.50 ^NS^	23.79 ± 3.90 ^NS^	LOD-55.5
Cotton	50	23 (46.0)	17.29 ± 2.40 **	20.25 ± 3.80 **	LOD-99.5	40	25 (62.5)	22.42 ± 3.50 **	25.31 ± 3.60 **	LOD-122.5
Grapeseed	45	25 (55.5)	6.25 ± 3.20 **	6.48 ± 4.30 **	LOD-33.5	55	30 (54.5)	12.17 ± 3.40 **	14.42 ± 3.70 **	LOD-110.5
Total	260	125 (48.1)				260	132 (50.7)			

^N.S^: the differences in aflatoxins levels between storage periods for edible seed oils were non-significant (*p* ≥ 0.05). ** the differences in aflatoxins levels between storage periods for edible seed oils were significant (*p* ≤ 0.05).

**Table 3:** Estimation of dietary intake for AFs in sunflower oil in local population from Punjab, Pakistan.

Category	Type	Central Punjab						Southern Punjab					
		Age Groups						Age Groups					
		16–22		23–32		≤33		16–22		23–32		≥33	
		Mean	Max.	Mean	Max.	Mean	Max.	Mean	Max.	Mean	Max.	Mean	Max.
≤6 Months Storage	Consumption kg/day	0.0027		0.0025		0.0041		0.0025		0.0027		0.049	
	AFs average amount (µg/kg)	10.81	78.5	10.81	78.5	10.81	78.5	10.81	78.5	10.81	78.5	10.81	78.5
	Dietary intake ng/kg/day	0.53	3.90	0.45	3.30	0.61	4.50	0.53	3.90	0.45	3.30	7.3	53.2
≥2 Years Storage	Consumption kg/day	0.0027		0.0025		0.0049		0.0025		0.0027		0.049	
	AFs average amount (µg/kg)	13.70	95.5	13.70	95.5	13.70	95.5	13.70	95.5	13.70	95.5	13.70	95.5
	Dietary intake ng/kg/day	0.74	5.10	0.57	4.00	0.86	6.00	0.68	4.70	1.65	11.50	8.55	59.60

## Data Availability

The data is available upon request.
